# Chiral Discrimination Mechanisms by Silylated-Acetylated Cyclodextrins: Superficial Interactions vs. Inclusion

**DOI:** 10.3390/ijms232113169

**Published:** 2022-10-29

**Authors:** Federica Balzano, Gloria Uccello-Barretta, Giuseppe Sicoli, Letizia Vanni, Alessandra Recchimurzo, Federica Aiello

**Affiliations:** 1 Department of Chemistry and Industrial Chemistry, University of Pisa, Via G. Moruzzi 13, 56124 Pisa, Italy; 2 Institute for Chemical and Physical Processes, National Research Council (CNR-IPCF), Via G. Moruzzi 1, 56124 Pisa, Italy

**Keywords:** NMR, diffusion coefficient, ROESY, association constant, chiral solvating agent, compound B, methyl 2-chloropropionate, Lipodex E

## Abstract

Cyclodextrin derivatives constitute a powerful class of auxiliary agents for the discrimination of apolar chiral substrates. Both host–guest inclusion phenomena and interactions with the derivatizing groups located on the surface of the macrocycle could drive the enantiodiscrimination; thus, it is important to understand the role that these processes play in the rational design of new chiral selectors. The purpose of this study is to compare via nuclear magnetic resonance (NMR) spectroscopy the efficiency of silylated-acetylated α-, β-, and γ-cyclodextrins in the chiral discrimination of 1,1,1,3,3-pentafluoro-2-(fluoromethoxy)-3-methoxypropane (compound B) and methyl 2-chloropropionate (MCP). NMR DOSY (Diffusion Ordered SpectroscopY) experiments were conducted for the determination of the bound molar fractions and the association constants, whereas ROESY (Rotating-frame Overhauser Enhancement SpectroscopY) measurements provided information on the hosts’ conformation and on the interaction phenomena with the guests. Compound B, endowed with fluorinated moieties, is not deeply included due to attractive Si-F interactions occurring at the external surface of the cyclodextrins. Therefore, a low selectivity toward the size of cyclodextrin cavity is found. By contrast, enantiodiscrimination of MCP relies on the optimal fitting between the size of the guest and that of the cyclodextrin cavity.

## 1. Introduction

The molecular basis of enantiorecognition processes originated by cyclodextrins (CDs) is not yet well understood despite the prominent role of this class of chiral auxiliaries in the field of the enantioselective chromatographic technologies [1,2,3]. In consideration of the quite elusive knowledge to this regard, a great deal of effort has been continuously addressed on the elucidation of the stereochemistry, dynamics, and thermodynamics of the diastereomeric complexes formed by cyclodextrins and enantiomeric substrates [4]. Such studies can be performed via spectroscopic investigations, mainly nuclear magnetic resonance (NMR) spectroscopy [5,6,7,8], together with computational methods [9,10,11,12].

Even though most of the studies dealing with native cyclodextrins point to the inclusion of the substrate into the hydrophobic cavity as the fundamental interaction mechanism on which chiral recognition relies [13,14,15,16], the origin of enantiodiscrimination by cyclodextrin derivatives is still the subject of controversial opinions [17,18].

Cyclodextrin derivatives endowed with silyl/alkanoyl or alkyl/alkanoyl derivatizing groups represent popular chiral auxiliaries for gas chromatographic applications in the area of the enantiodiscrimination of chiral substrates lacking in hydrogen bond donor groups, which constitute challenging systems for the majority of the enantioseparation methods [19,20,21]. As an example, the gas chromatographic separation of the two enantiomers of 1,1,1,3,3-pentafluoro-2-(fluoromethoxy)-3-methoxypropane (compound B), a chiral degradation product of the fluorinated anesthetic sevoflurane [22], was achieved with high efficiency by selecting heptakis(2,3-di-*O*-acetyl-6-*O*-*tert*-butyldimethylsilyl)-β-cyclodextrin (AcSiCD7) as the chiral agent [23]. Further NMR studies indicated that the guest is not deeply included into the CD cavity, but rather the enantiodiscrimination is driven by Si-F interactions [24]. Analogously, heptakis(3-*O*-butanoyl-2,6-di-*O*-*n*-pentyl)-β-cyclodextrin has been tested with this substrate, showing only moderate efficiency in its gas chromatographic enantiodiscrimination [25]. On the contrary, its γ-analog (Lipodex E), largely applied as chiral selector in chromatographic separations [26], proved to be remarkably efficient in differentiating the two enantiomers of compound B [25]. Interestingly, in this last case, subsequent NMR investigations pointed out a prominent role of the inclusion phenomenon in the chiral differentiation [27].

This outcome, apparently in conflict with the NMR results obtained with the silylated-acetylated β-CD [24], may be explained by keeping in mind that an optimal host–guest size fitting is not the only factor to be considered when selecting a CD as chiral agent. The complexing properties of CD derivatives, in particular, can be deeply influenced by the nature of the substituents, affecting the conformation of the host molecule and, hence, the shape of the cavity and its ability to include a substrate [18]. Moreover, the derivatizing groups can be directly involved in strong interactions with the substrates, driving toward mechanisms other than inclusion [24,28,29].

**Scheme 1:**
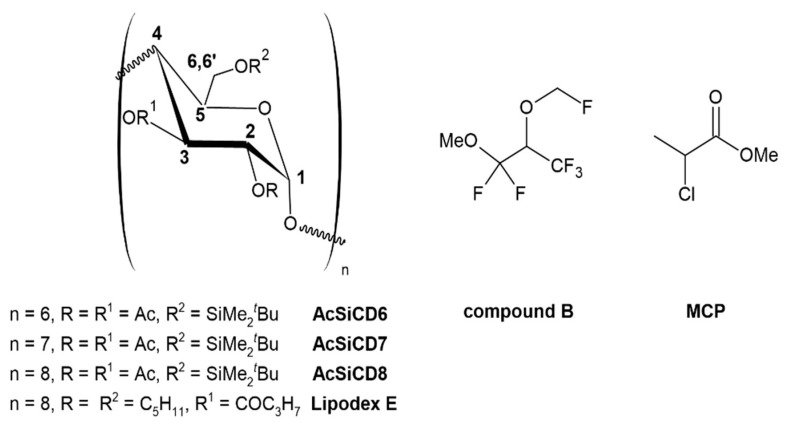
Chemical structures and numbering scheme for NMR analysis of the cyclodextrin derivatives involved in the study, and chemical structure of the two substrates compound B and methyl 2-chloropropionate.

In view of the above considerations, to ascertain the role of size cavity in the enantiodiscriminating efficiency, the three silylated-acetylated cyclodextrins (AcSiCD6, AcSiCD7, and AcSiCD8, Scheme 1) have been compared in the enantioselective interaction with compound B and methyl 2-chloropropionate (MCP, Scheme 1) via solution NMR spectroscopy in deuterated cyclohexane (C_6_D_12_), to better mimic the gas chromatographic environment. Compound B is endowed with fluorinated moieties able to interact with cyclodextrin silicon atoms at their external surface. MCP, frequently employed as probe in the analysis of the gas chromatographic behavior of cyclodextrin derivatives [30,31,32], is devoid of such functionalities.

The origin of chiral recognition has been carefully investigated by comparing thermodynamic and stereochemical features of the diastereomeric solvates formed in solution by exploiting Rotating-frame Overhauser Enhancement SpectroscopY (ROESY) and Diffusion Ordered SpectroscopY (DOSY) methods.

## 2. Results and Discussion

### 2.1. Conformation of the Cyclodextrin Derivatives

The conformational features of AcSiCD7 in deuterated cyclohexane (C_6_D_12_) have already been described in the literature [24]; therefore, in this work, the focus has been on the analysis of AcSiCD6 and AcSiCD8 conformation and on the comparison with AcSiCD7. 

ROESY experiments were exploited for investigating the occurrence of rotation of the glucopyranose rings about the glycosidic bonds (see Figure S1 in Supplementary Material), usually expected for cyclodextrin derivatives [33,34] and already observed for AcSiCD7 [24]. In the absence of rotation, the H1 proton (see Scheme 1) is closer to H4′ (the apex indicates the proton belonging to an adjacent ring) than H2, and ROE_1–2_ < ROE_1–4′_. If rotation is observed (ROE_1–2_ ≥ ROE_1–4′_), its direction can be established by the presence of the dipolar interaction of H1–H5′ (clockwise rotation) and/or H1–H3′ (anti-clockwise). Together with rotation, distortions of the glucopyranose rings can contribute to deviations from the truncated-cone shape typical of native CDs. These distortions can be detected by analyzing the dipolar effects generated by proton H2: when the ring is in a chair conformation, the proton mainly produces a ROE effect with the H1 and H4 nuclei, whereas in the case of distortions an additional effect with proton H3 is observed. In this case, a significant contribution of the skew conformation (Figure S1, in Supplementary Material) can be considered.

The 1D ROESY spectra, corresponding to the selective perturbation of H1 and H2 protons, are reported in Figure S2 in Supplementary Material. For both AcSiCD6 and AcSiCD8, the analysis revealed the occurrence of rotation about the glycosidic bond: the ROE effect detected for proton pair H1–H4′ has a lower intensity than that for the ROE H1–H2, and the dipolar effect observed between H1 and H5′ suggests a prevailing clockwise rotation. In addition, the presence of a ROE effect between H2 and H3 indicates distortion of the rings (skew). It is important to keep in mind that a similar conformation was found in C_6_D_12_ also for the β-derivative AcSiCD7 [24]. Therefore, in all the three cyclodextrins, deviations from the truncated-cone shape place the methyl protons bound to Si on the narrower rim near the acetyl groups lying on wider rim (Figure S3, Supplementary Material). It is noteworthy that not every kind of derivatizing group produces deviations from the truncated-cone shape; as a matter of fact, in the case of exhaustively derivatized Lipodex E (Scheme 1), no significant rotation or distortion was indicated, but rather self-inclusion of one of the pentyl moieties into the cyclodextrin cavity [35,36].

### 2.2. Enantiodiscrimination Processes by AcSiCDs

The enantiodiscriminating efficiency of the three silylated-acetylated cyclodextrins was investigated by analyzing equimolar mixtures CD/(*R*,*S*)-substrate in C_6_D_12_. For both compounds, non-equivalence (ǀδ*_R_* − δ*_S_*ǀ) and complexation shifts (Δδ = δ_mix_ − δ_free_) detected in the ^1^H NMR spectra were compared to assess the enantiodifferentiation ability of the different derivatives.

Knowing that the NMR enantiodiscrimination processes can be influenced both by stereochemical and thermodynamic factors, DOSY experiments were performed for calculating the bound molar fraction (x_b_) and the association constant (K) of each enantiomer of the two substrates. These parameters can be obtained, in fact, by measuring the translational diffusion coefficients of the single pure compounds alone and in their mixtures with CDs. 

The diffusion coefficient (D) measured for a compound can be correlated to its hydrodynamic radius (R_H_), and hence to the molecular sizes, by means of the Stokes–Einstein equation (Equation (1)):

D = kT/(6ηπR_H_) (1)

where k is the Boltzmann constant, T the absolute temperature, and η the viscosity of the solution. On the basis of Equation (1), the occurrence of complexation phenomena causes a decrease in the diffusion coefficient of the substrate, due to the increase in its apparent sizes. When fast exchange conditions apply, the measured diffusion coefficient (D_obs_) of every component in a mixture is the weighted average of its value in the free (D_f_) and in the bound (D_b_) states (Equation (2)):

D = D_f_x_f_ + D_b_x_b_ = D_f_ (1 − x_b_) + D_b_x_b_ (2)

where x_f_ and x_b_ are the molar fractions in the free and bound state, respectively.

In case of host–guest interaction with cyclodextrins, the diffusion of the complexed substrate is mainly driven by the macrocycle, whose diffusion coefficient is basically unaffected by the interaction with the small molecule. With these premises in mind, D_b_ can be approximated to that of the CD and the bound molar fraction can be easily calculated from Equation (2), thus allowing obtaining the association constant from Equation (3) by a single-point measurement [37]:

K = x_b_/(C − C_0_x_b_) (1 − x_b_) (3)

where C and C_0_ are the concentration of the host (cyclodextrin) and the guest (compound B or MCP), respectively.

#### 2.2.1. Compound B

**Figure 1:**
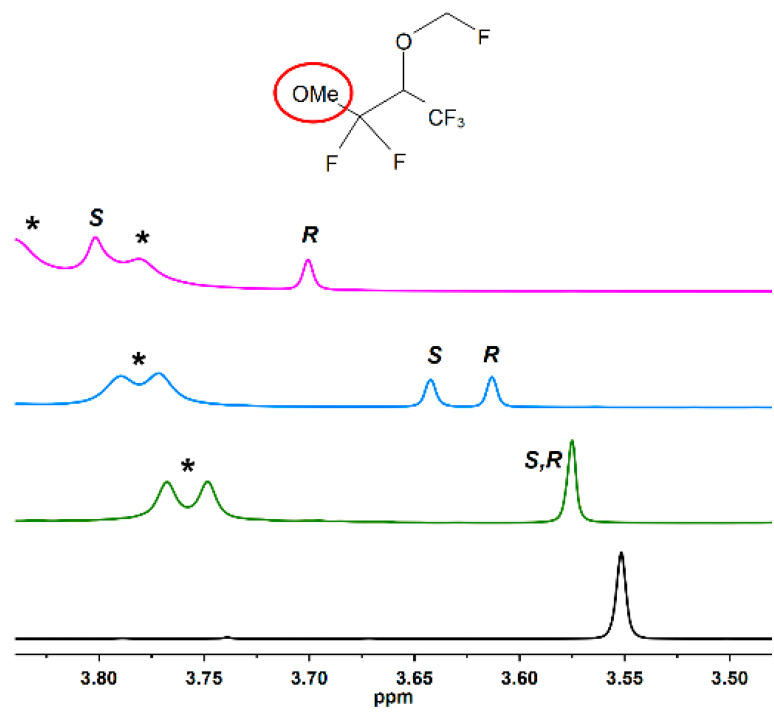
Expansions of ^1^H NMR (600 MHz, C_6_D_12_, 25 °C) spectra of (*R*,*S*)-compound B (60 mM) alone (black) and in 1:1 mixture with AcSiCD6 (green), AcSiCD7 (blue), and AcSiCD8 (magenta), respectively. * indicates CD signals.

The three silylated-acetylated cyclodextrins were tested with compound B at 60 mM concentration; the non-equivalences (ǀδ*_R_* – δ*_S_*ǀ) observed for the methoxy group, which resonates at 3.55 ppm in the pure compound (Figure 1), are reported in Table 1. 

It can be observed that AcSiCD6 does not differentiate at all the two enantiomers and causes only a small shift (3.57 ppm) at higher frequencies of the observed proton resonance. In the mixture containing the β-derivative AcSiCD7, the resonances of the two enantiomers are instead well differentiated and shifted at 3.64 ppm (δ*_S_*) and 3.61 ppm (δ*_R_*). Finally, with AcSiCD8, a remarkable increase in the non-equivalence is observed (0.101 ppm, Table 1) for the methoxy groups of the two enantiomers, which are also significantly shifted at higher frequencies (δ*_S_* = 3.80 ppm, δ*_R_* = 3.70 ppm). This trend highlights a better enantiodifferentiation efficiency of the larger CD as chiral selector for the fluorinated substrate.

The measurement of the diffusion parameters allowed calculation of the bound molar fractions and the association constants reported in Table 1. The diffusion data indicate that AcSiCD6, even if does not discriminate between the two enantiomers of compound B, causes a decrease in the diffusion coefficient (−5.03 × 10^−10^ m^2^/s, Table S1 in Supplementary Material), and this variation translates into a calculated bound molar fraction equal to 0.43. Such a significant percentage of x_b_ suggests that, even if there is no enantiodiscrimination, the smallest cyclodextrin is able to interact with the substrate. The amount of x_b_ calculated for this substrate remains almost unchanged for the (*R*)-enantiomer in the presence of both AcSiCD7 and AcSiCD8, to indicate a scarce selectivity toward the host to guest size fitting. An unwanted superimposition between the proton signals of compound B and AcDSi8 in the equimolar mixture did not allow the measurement of the diffusion coefficient for both enantiomers but, on the basis of the complexation shift trend (Table 1), it can be reliably assessed that the (*S*)-enantiomer is tightly bound to AcSiCD8, analogously to the case of AcSiCD7 [24]. It is noteworthy that in the previous investigation dealing with the interaction of the single enantiomers of compound B and AcSiCD7, the association constants calculated for the two diastereomeric solvates were very similar to those directly obtained for the racemate, thus confirming that no competition between the two enantiomers in the interaction with the chiral selector occurs [24]. Interestingly, the fact that the association constant of the diastereomeric solvates scarcely depends on the cavity size, whereas non-equivalence is remarkably sensitive to it, strongly suggests that enantiodifferentiation mainly depends on the number of glucopyranose units.

**Figure 2:**
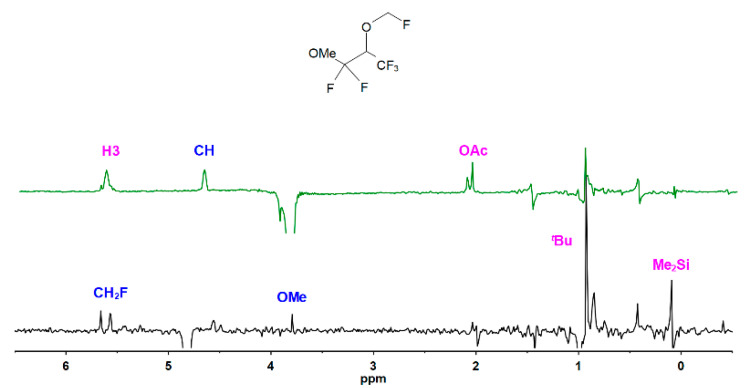
2D ROESY (600 MHz, C_6_D_12_, 25 °C, mixing time = 600 ms) traces of the methine (black) and OMe (green) protons of compound B (60 mM) in a 1:1 mixture with AcSiCD8. The magenta protons belong to the host, the blue to the guest.

The analysis of the ROESY map recorded for the mixture compound B/AcSiCD8 pointed out a dipolar interaction between the methoxy group of compound B and proton H3 of AcSiCD8 lying on its large rim (Figure 2), highlighting that inclusion is not deep. Unexpectedly, an inter-molecular ROE effect is detected between the methine proton of the guest and the *tert*-butyldimethylsilyl groups of the cyclodextrin (Figure 2). Such an intermolecular interaction is possible only by virtue of the above discussed relevant rotation (Section 2.1) about the glycosidic linkages that brings the protruding fluorinated moieties of compound B in the proximity of the silyl groups, probably due to the attractive Si-F interactions.

#### 2.2.2. Methyl 2-Chloropropionate

**Figure 3:**
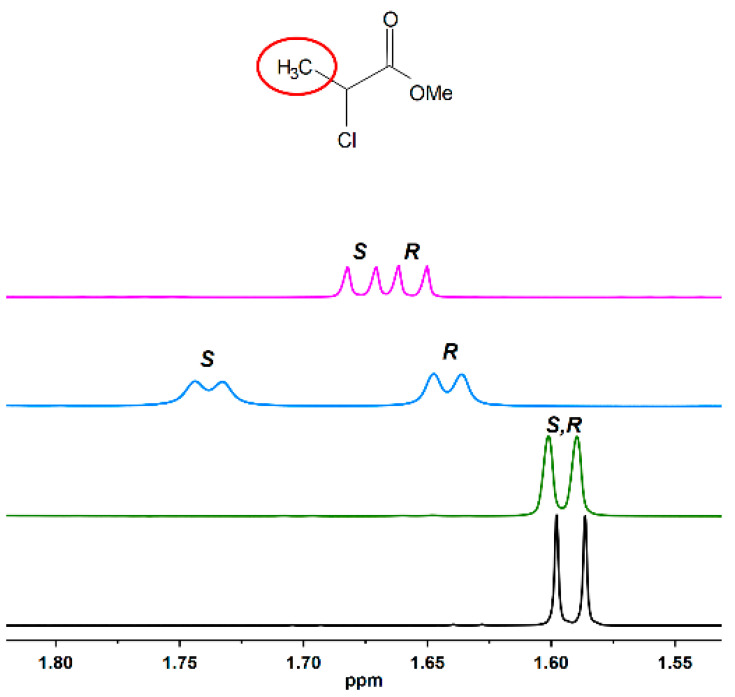
Expansions of ^1^H NMR (600 MHz, C_6_D_12_, 25 °C) spectra of (*R*,*S*)-MCP (20 mM) alone (black) and in a 1:1 mixture with AcSiCD6 (green), AcSiCD7 (blue), and AcSiCD8 (magenta), respectively.

The three cyclodextrins were then tested with the second chiral substrate, MCP. Data show that, analogously to what was observed for compound B, AcSiCD6 does not generate enantiodiscrimination of the methyl protons of the two enantiomers (Figure 3 and Table 2). The 7- and 8-membered ring derivatives are able to differentiate the two enantiomers also in this case; however, interestingly, AcSiCD7 proved to be the best chiral selector for MCP in terms of enantioseparation. A significant non-equivalence was measured for the protons of the substrate, in particular for the methine one (0.218 ppm, Table S2), in the presence of this chiral selector.

Regarding the analysis of diffusion coefficients, the highest variations between the diffusion coefficients of the two enantiomers are observed when the compound is mixed with the β-derivative (Table S3). In this case, at 20 mM, already more than half of (*S*)-MCP is bound (x_b_ = 0.57, Table 2), while (*R*)-MCP interacts to a lesser extent (x_b_ = 0.34). Interestingly, in the mixture with AcSiCD8 this difference is lost, and the bound molar fractions calculated for the two enantiomers are comparable and lower with respect to those obtained in the presence of the 7-membered ring cyclodextrin (Table 2). We can speculate that the increased cavity size affects the thermodynamics of the enantiodiscrimination process, which is then only related to stereochemical differentiation. With AcSiCD7, the good fit between the cavity of the macrocycle and the substrate favors the interaction and allows the formation of stable host–guest complexes, with a preference for the (*S*)-enantiomer. 

**Figure 4:**
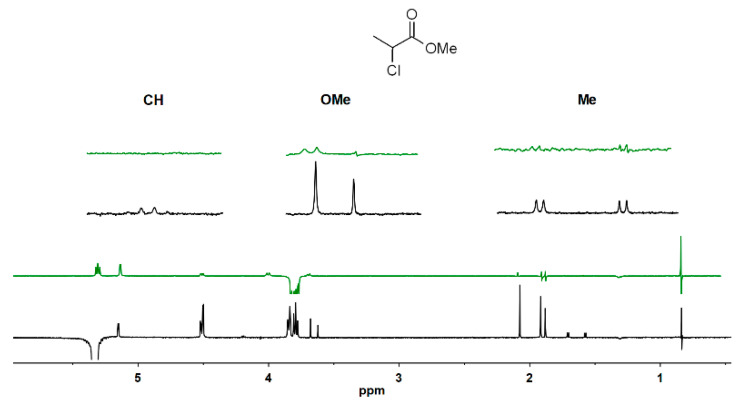
1D ROESY (600 MHz, C_6_D_12_, 25 °C, mixing time = 600 ms) spectra of H3 (bottom, black) and H5 (top, green) protons of AcSiCD7 in a 1:1 mixture with MCP (20 mM).

Inclusion of MCP inside the AcSiCD7 cavity was verified by analyzing the 1D ROESY spectra, where dipolar interactions were detected between MCP protons and both internal protons H3 and H5 of the cyclodextrin (Figure 4).

The comparison between the ROE spectra recorded for H3 and H5 protons points at an inclusion mechanism that takes place from the larger rim of the host, since stronger ROE effects are detected between MCP protons and H3 with respect to H5, which is located on the smaller part of the cavity.

## 3. Materials and Methods

### 3.1. Materials

Hexakis(2,3-di-*O*-acetyl-6-*O*-*tert*-butyldimethylsilyl)-α-cyclodextrin (AcSiCD6), heptakis(2,3-di-*O*-acetyl-6-*O*-*tert*-butyldimethylsilyl)-β-cyclodextrin (AcSiCD7), octakis(2,3-di-*O*-acetyl-6-*O*-*tert*-butyldimethylsilyl)-γ-cyclodextrin (AcSiCD8), and 1,1,1,3,3-pentafluoro-2-(fluoromethoxy)-3-methoxypropane (compound B) were kindly provided by Professor V. Schurig; methyl 2-chloropropionate (MCP) was purchased from Merck (Darmstadt, Germany); C_6_D_12_ was purchased from Deutero GmbH (Kastellaun, Germany). All chemicals were used without further purification.

### 3.2. Methods

^1^H NMR measurements were carried out in C_6_D_12_ on a Varian (Palo Alto, CA, USA) INOVA600 spectrometer equipped with a 5 mm probe operating at 600 MHz for ^1^H nuclei. The proton chemical shifts are relative to tetramethylsilane (TMS) as the secondary reference standard, and the temperature was controlled (25 ± 0.1 °C). For the 2D NMR spectra, the spectral width used was the minimum required in both dimensions. The 2D-ROESY maps were recorded using a relaxation time of 8 s and a mixing time of 600 ms; 512 increments of 4 transients of 2 K points each were collected. The 1D-ROESY spectra were recorded using a selective inversion pulse, 2048 transients, a relaxation delay of 1 s, and a mixing time of 300 or 600 ms. DOSY experiments were performed using a stimulated echo sequence with self-compensating gradient schemes and 64 K data points. Typically, g was varied in 15 or 20 steps (8 transients each) and Δ and δ were optimized to obtain an approximately 90−95% decrease in the resonance intensity at the largest gradient amplitude. The baselines of all arrayed spectra were corrected prior to processing the data. After data acquisition, each FID was apodized with 1.0 Hz line broadening and Fourier transformed. The data were processed with the DOSY macro (involving the determination of the resonance heights of all of the signals above a pre-established threshold and the fitting of the decay curve for each resonance to a Gaussian function) to obtain pseudo-two-dimensional spectra with NMR chemical shifts along one axis and calculated diffusion coefficients along the other. NMR characterization data are reported in the Supplementary Material.

## 4. Conclusions

On the basis of the above-described findings, it can be acknowledged that interactions at the external surface of derivatized cyclodextrins may drive the chiral recognition mechanisms mainly when deviations from the truncated-cone shape of the host occur. In the case of supramolecular complexes formed between compound B and silylated-acetylated cyclodextrins, intermolecular attractive Si-F interactions may occur by virtue of the relevant rotation about the glycosidic linkages. A higher enantiodiscrimination for compound B is obtained in the presence of AcSiCD8 than in the presence of AcSiCD7, despite comparable values of the association constants, at least for the (*R*)-enantiomer. Therefore, enantiodiscrimination seems to be more correlated to the number of glucopyranose units than it is to the cavity size.

By contrast, AcSiCD7 in comparison with AcSiCD8, not only better differentiates MCP enantiomers that are devoid of fluorine atoms, but also the calculated association constants are larger for the β-cyclodextrin derivative, showing a strong dependence on the optimal host to guest size fitting. MCP inclusion is deeper than it is for compound B, according to the prevailing inclusion mechanism, where relevant intermolecular interactions at the external surface of the macrocycle compete with a deep inclusion.

Interestingly, previously reported data regarding the analysis of the interaction mechanisms between compound B and Lipodex E, and between compound B and the corresponding β-derivative, both lacking in silylated groups, demonstrated that the strength of interaction depends exclusively on the optimal fit between the cyclodextrin cavity and the guest [27].

Finally, it is important to highlight that, despite the fact that the investigation into the interaction mechanisms involving cyclodextrin derivatives are complicated by the symmetry of the macrocycles, NMR spectroscopy allows, by combining DOSY and ROESY experiments, to obtain valuable information on the interaction mechanism on which chiral recognition relies.

## Figures and Tables

**Table 1:** Equimolar mixtures of compound B (60 mM) with AcSiCD6, AcSiCD7, or AcSiCD8 (600 MHz, C_6_D_12_, 25 °C): non-equivalences (ǀδ*_R_* − δ*_S_*ǀ, ppm) and complexation shifts (Δδ = δ_mix_ – δ_free_, ppm) measured for the methoxy group of compound B; bound molar fractions (x_b_) and association constants (K, M^−1^) obtained from diffusion data.

CD	^1^H NMR Analysis (OMe)		Diffusion Measurements	
	|δ*_R_* − δ*_S_*ǀ	Δδ	x_b_	K
AcSiCD6	0	0.023	0.43	22.1 ± 7.9
AcSiCD7	0.026	0.089 (*S*)	0.75 (*S*)	78.8 ± 32.6 (*S*)
		0.063 (*R*)	0.68 (*R*)	55.1 ± 19.6 (*R*)
AcSiCD8	0.101	0.250 (*S*)	n.d. ^1^	n.d. ^1^
		0.149 (*R*)	0.64 (*R*)	42.7 ± 13.7 (*R*)

^1^ Not determined due to strong superimposition between compound B and CD signals.

**Table 2:** Equimolar mixtures of MCP (20 mM) with AcSiCD6, AcSiCD7, or AcSiCD8 (600 MHz, C_6_D_12_, 25 °C): non-equivalences (ǀδ*_R_* − δ*_S_*ǀ, ppm) and complexation shifts (Δδ = δ_mix_ – δ_free_, ppm) measured for the methyl group of MCP; bound molar fractions (x_b_) and association constants (K, M^−1^) obtained from diffusion data.

CD	^1^H NMR Analysis (OMe)		Diffusion Measurements	
	|δ*_R_* − δ*_S_*ǀ	Δδ	x_b_	K
AcSiCD6	0	0.002	0.03	/
AcSiCD7	0.096	0.151 (*S*)	0.57 (*S*)	92.8 ± 24.5 (*S*)
		0.054 (*R*)	0.34 (*R*)	30.6 ± 7.6 (*R*)
AcSiCD8	0.020	0.089 (*S*)	0.26 (*S*)	20.6 ± 8.1 (*S*)
		0.069 (*R*)	0.25 (*R*)	19.0 ± 8.4 (*R*)

## Data Availability

Not applicable.

## Supplementary Materials

The supporting information can be downloaded at: https://www.mdpi.com/article/10.3390/ijms232113169/s1.
