# Supplementary material for: Chiral Discrimination Mechanisms by Silylated-Acetylated Cyclodextrins: Superficial Interactions vs. Inclusion
Source: Int J Mol Sci. 2022 Oct 29;23(21):13169. doi: 10.3390/ijms232113169 (PMC9656310; doi:10.3390/ijms232113169)
Supplement: Supplementary file 1 [file ijms-23-13169-s001.zip › ijms-1973572-supplementary.pdf]

Chiral discrimination mechanisms by silylated-acetylated cyclodextrins: superficial interactions *vs* inclusion

Federica Balzano [1], Gloria Uccello-Barretta [1,*], Giuseppe Sicoli [1,‡], Letizia Vanni [1], Alessandra Recchimurzo [1] and Federica Aiello [2,*]

[1] Department of Chemistry and Industrial Chemistry, University of Pisa, via G. Moruzzi 13, 56124 Pisa, Italy
[2] National Research Council, Institute for Chemical and Physical Processes (CNR-IPCF), via G. Moruzzi, 1, 56124 Pisa, Italy
‡ current address: Laboratoire Avancé de Spectroscopie pour les Interactions, la Réactivité et l'Environnement (LASIRE), Avenue Paul Langevin Bâtiment C4 – UMR CNRS 8516, Université de Lille, Sciences et Technologies, 59655 Villeneuve-d'Ascq Cedex, France;
* Correspondence: gloria.uccello.barretta@unipi.it; 00390502219232 (G.U.B.); federica.aiello@cnr.it; 00390503152265 (F.A.)

Supplementary Material

Table of contents

- Figure S1- Conformational features of cyclodextrins: (A) no rotation, (B) clockwise rotation, (C) anti-clockwise rotation, (D) skew distortion.

- Figure S2. AcSiCD6 (left) and AcSiCD8 (right): ^{1}H NMR (600 MHz, C_6D_{12}, 25 °C) spectra (black) and 1D ROESY (mixing time = 300 ms) spectra of H1 (red) and H2 (blue) protons; * indicates the solvent.

- Figure S3- 1D ROESY (600 MHz, C_6D_{12}, 25 °C, mixing time = 300 ms) spectra of methyl protons of AcSiCD6 (bottom, red), AcSiCD7 (center, green) and AcSiCD8 (top, blu).

- Table S1. Diffusion data (600 MHz, C_6D_{12}, 25 °C) obtained for compound B (60 mM) in equimolar mixtures with AcSiCD6, AcSiCD7 and AcSiCD8 (D_{obs} = diffusion coefficient measured in the mixture substrate/CD; D_f = diffusion coefficient of the substrate in the free state; $D_b \cong D_{CD}$).

- Table S2. Equimolar mixtures of MCP (20 mM) with AcSiCD6, AcSiCD7 and AcSiCD8 (600 MHz, C_6D_{12}, 25 °C): non-equivalences ($|\delta_R - \delta_S|$, ppm) and complexation shifts ($\Delta\delta = \delta_{mix} - \delta_{free}$, ppm).

- Table S3. Diffusion data (600 MHz, C_6D_{12}, 25 °C) obtained for MCP (20 mM) in equimolar mixtures with AcSiCD6, AcSiCD7 and AcSiCD8 (D_{obs} = diffusion coefficient measured in the mixture substrate/CD; D_f = diffusion coefficient of the substrate in the free state; $D_b \cong D_{CD}$).

- NMR characterization data of: AcSiCD6, AcSiCD7, AcSiCD8, compound B, MCP.

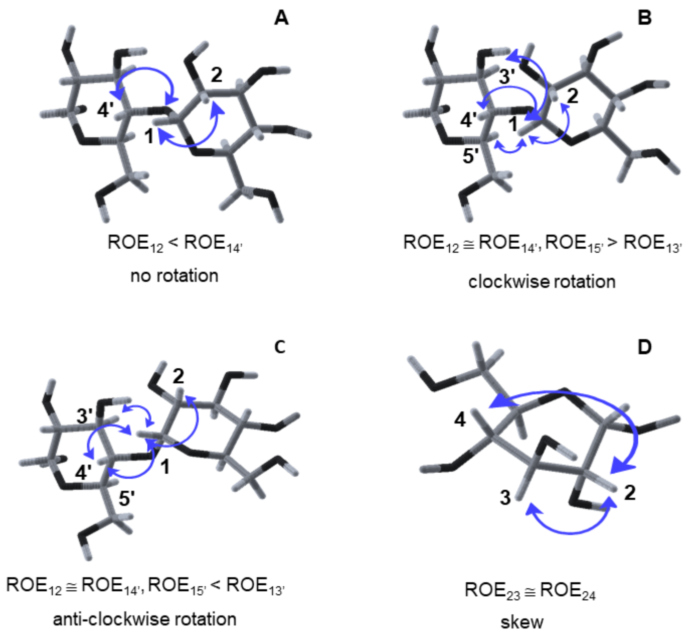

Figure S1- Conformational features of cyclodextrins: (A) no rotation, (B) clockwise rotation, (C) anti-clockwise rotation, (D) skew distortion.

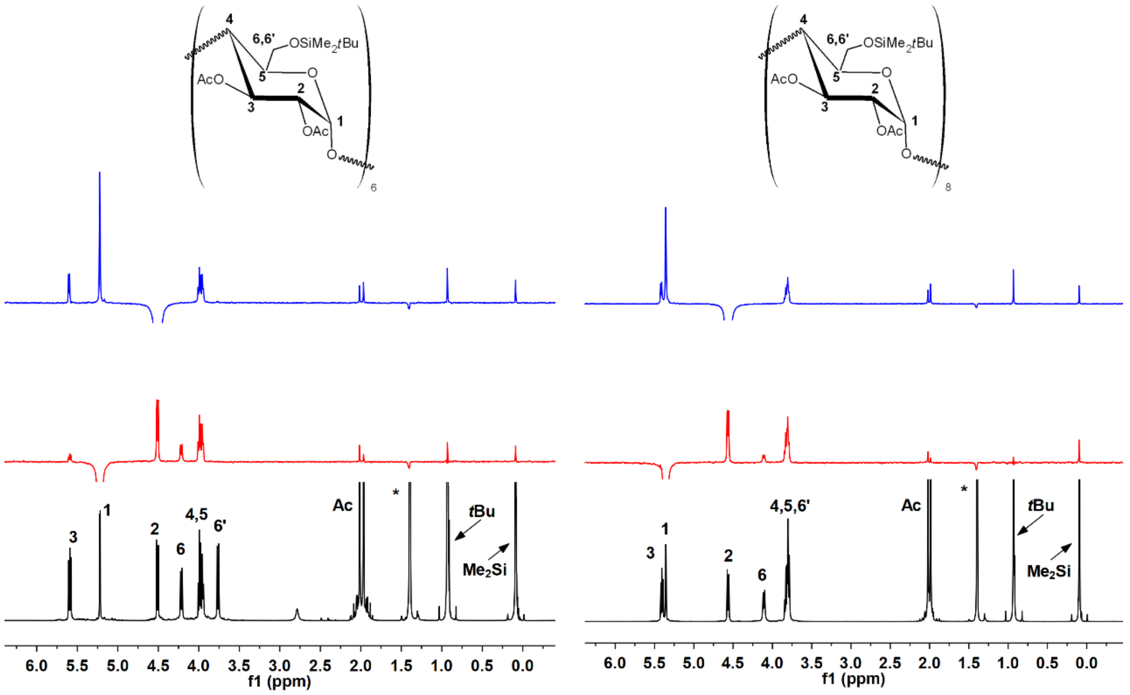

Figure S2. AcSiCD6 (left) and AcSiCD8 (right): ^{1}H NMR (600 MHz, C_6D_{12}, 25 °C) spectra (black) and 1D ROESY (mixing time = 300 ms) spectra of H1 (red) and H2 (blue) protons; * indicates the solvent.

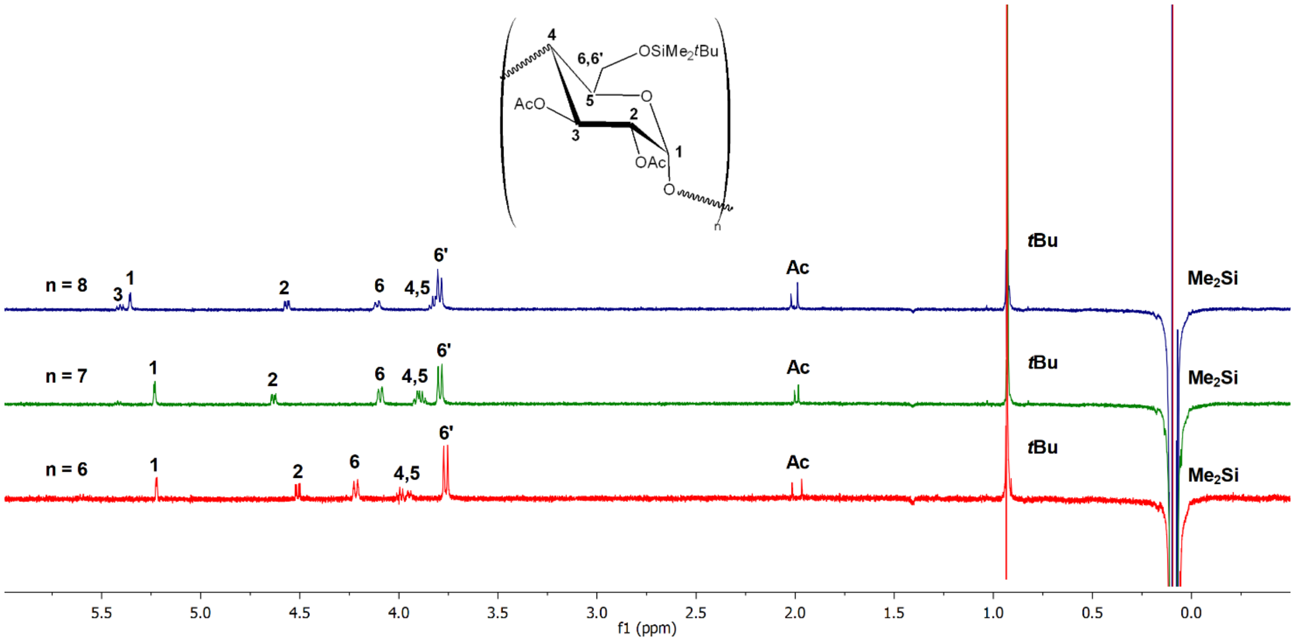

Figure S3- 1D ROESY (600 MHz, C_6D_{12}, 25 °C, mixing time = 300 ms) spectra of methyl protons of AcSiCD6 (bottom, red), AcSiCD7 (center, green) and AcSiCD8 (top, blu).

Table S1. Diffusion data (600 MHz, C_6D_{12}, 25 °C) obtained for compound B (60 mM) in equimolar mixtures with AcSiCD6, AcSiCD7 and AcSiCD8 (D_{obs} = diffusion coefficient measured in the mixture substrate/CD; D_f = diffusion coefficient of the substrate in the free state; $D_b \cong D_{CD}$).

**CD**	***Diffusion measurements***	
	$D_{obs} - D_f$	$D_b - D_f$
AcSiCD6	-5.03 ± 0.47	-11.58 ± 0.51
AcSiCD7	-8.94 ± 0.43 (*S*)	- 11.96 ± 0.51(*S*)
	-8.19 ± 0.44 (*R*)	-11.96 ± 0.51 (*R*)
AcSiCD8	n.d.[a] (*S*)	n.d.[a] (*S*)
	-7.67 ± 0.43 (*R*)	- 12.06 ± 0.51(*R*)

[a] not determined due to strong superimposition of compound B and CD signals.

Table S2. Equimolar mixtures of MCP (20 mM) with AcSiCD6, AcSiCD7 and AcSiCD8 (600 MHz, C_6D_{12}, 25 °C): non-equivalences ($|\delta_R - \delta_S|$, ppm) and complexation shifts ($\Delta\delta = \delta_{mix} - \delta_{free}$, ppm).

**CD**	**MCP**			
	CH		***OMe***	
	$|\delta_R - \delta_S|$	$\Delta\delta$	$|\delta_R - \delta_S|$	$\Delta\delta$
AcSiCD6	0	0.004	0	0.007
AcSiCD7	0.218	0.285 (*S*)	0.044	0.089 (*S*)
		0.067 (*R*)		0.045 (*R*)
AcSiCD8	0.070	0.250 (*S*)	0.020	0.107 (*S*)
		0.180 (*R*)		0.087 (*R*)

Table S3. Diffusion data (600 MHz, C_6D_{12}, 25 °C) obtained for MCP (20 mM) in equimolar mixtures with AcSiCD6, AcSiCD7 and AcSiCD8 (D_{obs} = diffusion coefficient measured in the mixture substrate/CD; D_f = diffusion coefficient of the substrate in the free state; $D_b \cong D_{CD}$).

**CD**	***Diffusion measurements***	
	D_{obs} - D_f	D_b - D_f
AcSiCD6	-0.43 ± 0.88	-15.90 ± 0.61
AcSiCD7	-9.18 ± 0.58 (*S*)	-16.10 ± 0.61(*S*)
	-5.43 ± 0.54 (*R*)	-16.10 ± 0.61 (*R*)
AcSiCD8	-4.30 ± 0.96(*S*)	-16.30 ± 0.61(*S*)
	-4.07 ± 0.95 (*R*)	-16.30 ± 0.61(*R*)

NMR characterization data

AcSiCD6 (n = 6)

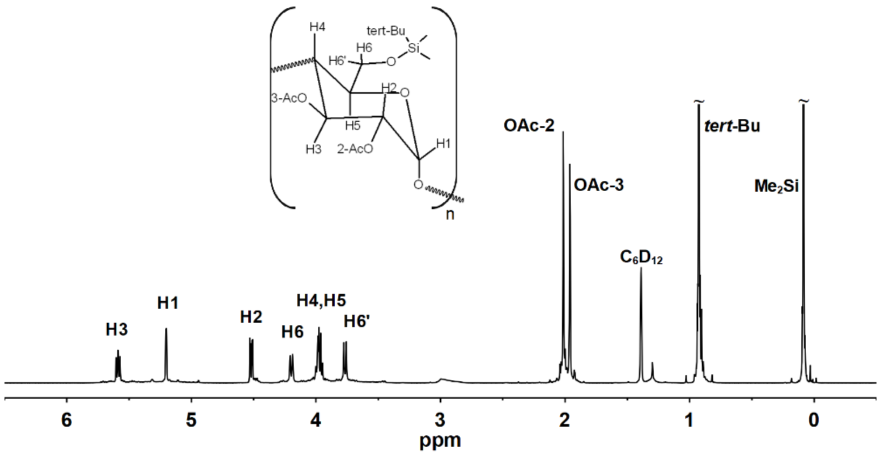

^{1}H NMR (600 MHz, C_6D_{12}, 25 °C) δ (ppm): 5.59 (6H, H3, dd, J_{32} = 10.3 Hz, J_{34} = 8.5 Hz), 5.20 (6H, H1, d, J_{12} = 3.2 Hz), 4.50 (6H, H2, dd, J_{23} = 10.3 Hz, J_{21} = 3.2 Hz), 4.20 (6H, H6, br d, $J_{66'}$ = 12.4 Hz), 3.98 (6H,

H5), 3.86 (6H, H4, dd, $J_{43} = J_{45} = 8.5$ Hz), 3.77 (6H, H6′, br d, $J_{6'6} = 12.4$ Hz), 2.01 (18H, Ac-2, s), 1.96 (18H, Ac-3, s), 0.93 (54H, *tert*-Bu, s), 0.09 (36H, Me_2Si, s).

^{13}C NMR (150 MHz, C_6D_{12}, 25 °C) δ (ppm): 169.2 (CO, Ac-3), 169.0 (CO, Ac-2), 95.9 (C1), 74.8 (C4), 72.1 (C5), 71.8 (C3), 71.5 (C2), 62.5 (C6), 25.7 (($CH_3)_3C$), 20.3 (Me, Ac-2), 19.7 (Me, Ac-3), 18.3 (($CH_3)_3C$), -5.7 and -5.4 ((Me)$_2$Si).

AcSiCD7 (n=7)

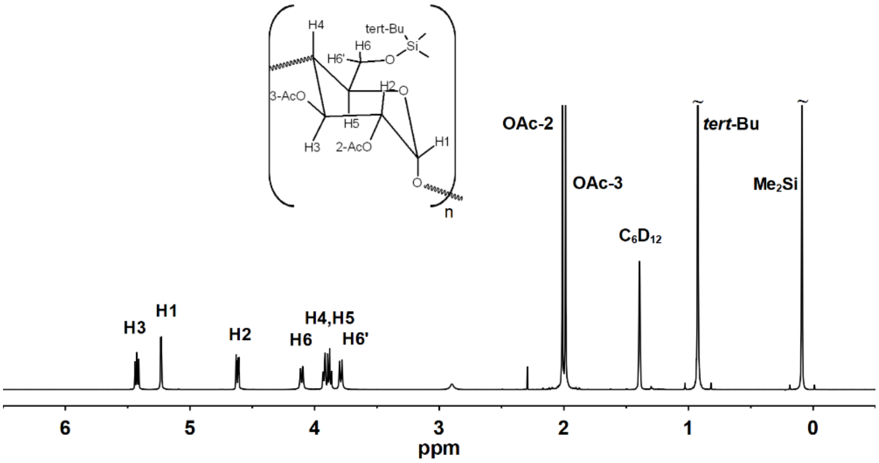

^{1}H NMR (600 MHz, C_6D_{12}, 25 °C) δ (ppm): 5.43 (7H, H3, dd, $J_{32} = 9.9$ Hz, $J_{34} = 8.6$ Hz), 5.23 (7H, H1, d, $J_{12} = 3.7$ Hz), 4.62 (7H, H2, dd, $J_{23} = 9.9$ Hz, $J_{21} = 3.7$ Hz), 4.10 (7H, H6, br d, $J_{66'} = 10.0$ Hz), 3.93 (7H, H5, br d, $J_{54} = 9.6$ Hz), 3.88 (7H, H4, dd, $J_{45} = 9.6$ Hz, $J_{43} = 8.6$ Hz), 3.79 (7H, H6′, br d, $J_{6'6} = 10.0$ Hz), 2.01 (21H, Ac-2, s), 1.99 (21H, Ac-3, s), 0.93 (63H, *tert*-Bu, s), 0.09 (42H, Me_2Si, s).

^{13}C NMR (150 MHz, C_6D_{12}, 25 °C) δ (ppm): 169.3 (CO, Ac-3), 168.9 (CO, Ac-2), 96.7 (C1), 75.6 (C4), 72.0 (C5), 71.5 (C2+C3), 62.3 (C6), 25.7 (($CH_3)_3C$), 20.3 (Me, Ac-2), 20.0 (Me, Ac-3), 18.2 (($CH_3)_3C$), -5.6 and -5.3 (($CH_3)_2$Si).

AcSiCD8 (n=8)

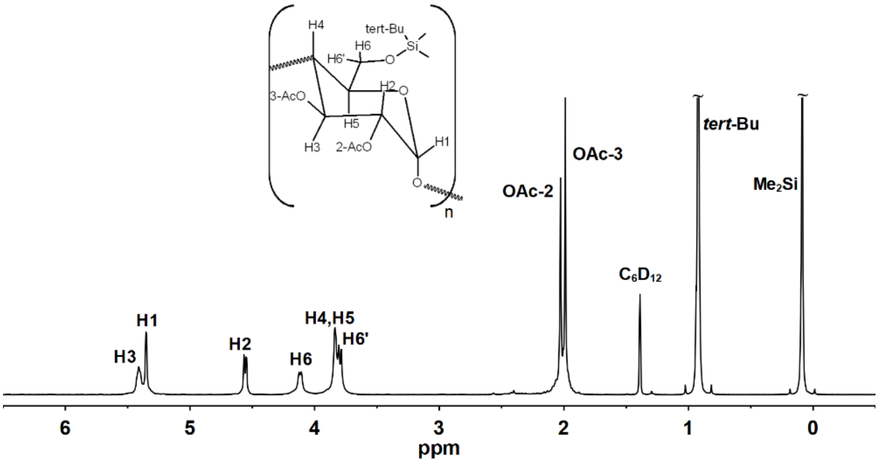

^{1}H NMR (600 MHz, C_6D_{12}, 25 °C) δ (ppm): 5.41 (8H, H3, br t, $J_{32} = J_{34} = 10.0$ Hz), 5.35 (8H, H1, br d, J_{12} = 3.4 Hz), 4.56 (8H, H2, dd, $J_{23} = 10.0$ Hz, $J_{21} = 3.4$ Hz), 4.11 (8H, H_6, br d, $J_{66'} = 11.4$ Hz), 3.84 (16H,

H4/H5, m), 3.79 (8H, $H_{6'}$, br d, $J_{6'6}$ = 11.4 Hz), 2.03 (24H, [CH3] Ac-2, s), 1.99 (24H, [CH3] Ac-3, s), 0.92 (72H, *tert*-Bu, s), 0.09 (48H, Me_2Si, s).

^{13}C NMR (150 MHz, C_6D_{12}, 25 °C) δ (ppm): 169.2 (CO, Ac-3), 169.0 (CO, Ac-2), 95.9 (C1), 74.4 (C4), 72.1 (C5), 70.8 (C2), 71.9 (C3), 62.1 (C6), 25.7 ($(CH_3)_3C$), 20.4 (Me, Ac-2), 19.9 (Me, Ac-3), 18.3 ($(CH_3)_3C$), -5.6 and -5.3 (Me_2Si).

Compound B

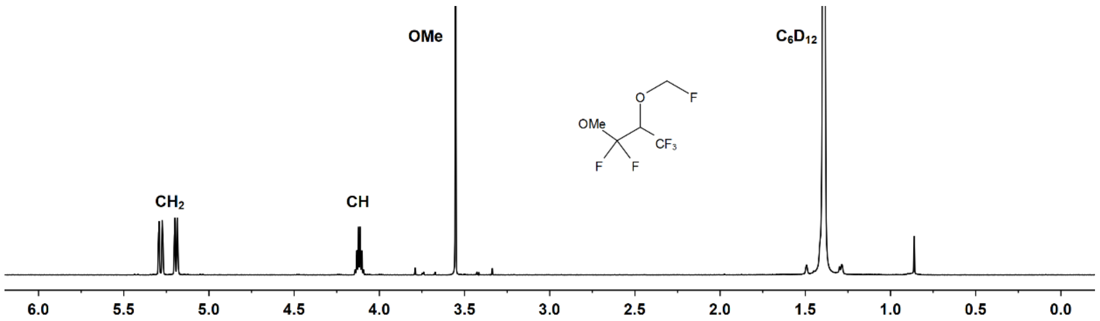

^1H NMR (600 MHz, C_6D_{12}, 25 °C) δ (ppm): 5.29 (2H, CH_2, dd, J_{HF} = 12.5 Hz, $J_{HH'}$ = 2.7 Hz), 5.21 (1H, CH2, dd, J_{HF} = 9.7 Hz, $J_{HH'}$ = 2.7 Hz), 4.13 (1H, CH, m), 3.55 (3H, MeO, s).

MCP

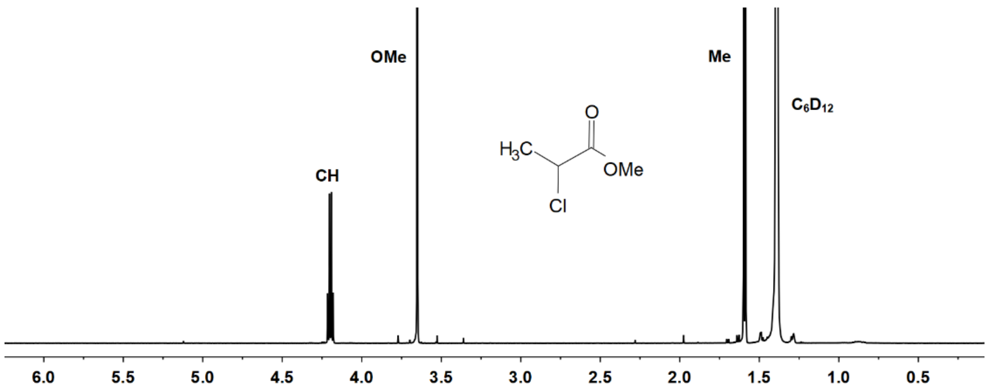

^1H NMR (600 MHz, C_6D_{12}, 25 °C) δ (ppm): 4.20 (1H, CH, q, J_{CH-Me} = 6.9 Hz), 3.65 (3H, OMe, s), 1.59 (3H, Me, d, J_{Me-CH} = 6.9 Hz).
